# The balanced and introspective brain

**DOI:** 10.1098/rsif.2016.0994

**Published:** 2017-05-31

**Authors:** P. A. Robinson

**Affiliations:** 1 School of Physics, University of Sydney, Sydney, New South Wales 2006, Australia; 2 Center for Integrative Brain Function, University of Sydney, Sydney, New South Wales 2006, Australia

**Keywords:** brain dynamics, neural field theory, criticality, balance, transfer functions

## Abstract

Transfers of large-scale neural activity into, within and between corticothalamic neural populations and brain hemispheres are analysed using time-integrated transfer functions and state parameters obtained from neural field theory for a variety of arousal states. It is shown that the great majority of activity results from feedbacks within the corticothalamic system, including significant transfer between hemispheres, but only a small minority arises via net input from the external world, with the brain thus in a near-critical, highly introspective state. Notably, the total excitatory and inhibitory influences on cortical neurons are balanced to within a few per cent across arousal states. Strong negative intrahemispheric feedforward exists to the cortex, and even larger interhemispheric positive feedforward, but these are modified by feedback loops to yield near-critical positive overall gain. The results underline the utility of transfer functions for the analysis of brain activity.

## 1. Introduction

Work over the last two decades has shown that the great majority of spikes that reach a given cortical neuron are not the result of direct input, nor even a direct chain of feedforward relays from the outside world, but are largely the outcome of recirculation of activity within the brain that leads to a near-critical state in humans, as confirmed by electroencephalography (EEG) of up to 1500 subjects and by functional magnetic resonance imaging (fMRI) [1–11]. Similar signs of criticality have been seen in spike avalanches in neural cell cultures [12] and non-human animals [13]. Excitatory and inhibitory gains have also been widely argued to be in approximate balance on theoretical grounds that such a state should enhance sensitivity to incoming signals [14], improve the efficiency of neural coding at the microscale [15,16] and enable complex dynamics [17]. Notably, near balance of excitation and inhibition can only occur near a critical point if the time-integrated (positive) excitatory and (negative) inhibitory gains for activity reaching cortical neurons are large in magnitude and sum to approximately unity.

Physiologically based neural field theory (NFT) is well suited to analysis of large-scale brain dynamics, including inference of gains. In particular, it has yielded gain values for a range of arousal states through fits of its predictions to experimental EEG spectra [1–3,7] and fits of its predictions of activity eigenmodes and eigenvalues to fMRI data [6,8]. For example, more than a decade ago it was estimated that in the alert eyes-open state cortical excitatory neurons have net positive gains of approximately +6.8 from others of the same type, +2.2 via thalamus and −8.1 from inhibitory cortical neurons, implying near balance at a near-critical state with an overall net gain of around +0.9 [1]. Our various studies since estimated net gains of 0.84–0.88, based on a variety of EEG and fMRI studies [1–3,5,8]. Our recent fMRI-based studies of the bihemispheric brain have yielded parameters consistent with these values, showing net gains of approximately 0.73 from intrahemispheric influences and 0.15 from interhemispheric connections (0.88 total), and 0.12 feedforward from outside the corticothalamic system [6,8].

The aim of this work is to further quantify overall balance and criticality in the corticothalamic system in various states of arousal, which provide the background against which finer-scale dynamic processes occur to support cognition and motor outputs, for example. Balance and criticality have not been brought together systematically, although fitted parameters have been published that enable the present analysis. Nor has there been a systematic study from this perspective of how influences propagate through feedforward, feedback and internal loops in the corticothalamic system that is responsible for most EEG, MEG and fMRI signals studied in neuroimaging. Hence, in §2 we use transfer functions to explore how activity propagates through the bihemispheric corticothalamic system, from external inputs to various populations, along both direct and indirect paths. This allows us to obtain expressions for feedforward and total gains in the system. In §3, we use transfer functions to analyse criticality and balance in various arousal states whose physiological parameters have been previously estimated. The implications of the results for balance, criticality and introspection in normal brain states are summarized and discussed in §4.

## 2. Theory

In this work, we assume that the normal dynamics of the brain at scales of around 1 mm and above involve approximately linear perturbations from a fixed point, an approximation that has yielded numerous experimentally validated predictions via NFT (see [2,18,19] and references cited therein). Using NFT, we derive expressions for the time-integrated transfer functions between populations that express the total influence without regard to timing. This is the appropriate measure for overall brain states and stability, although frequency-dependent transfer functions will be of interest in future work, as outlined in §5.

**Figure 1.**
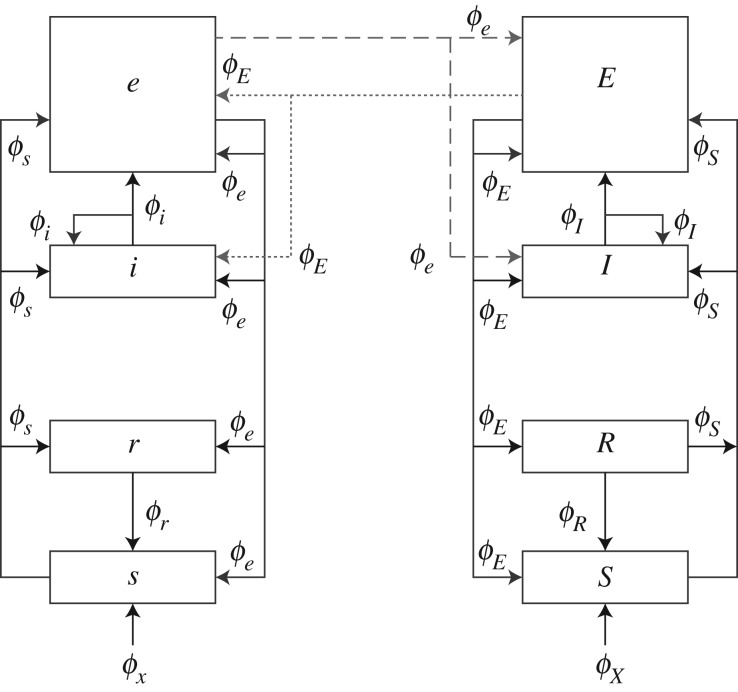
Schematic bihemispheric corticothalamic system showing activity fields *ϕ*_*a*_ emerging from left hemisphere populations and *ϕ*_*A*_ from right hemisphere populations, with corresponding synaptic gains *G*_*ab*_ as shown. Connections between populations are shown as arrows. Populations represent cortical excitatory pyramidal (*e*, *E*), cortical inhibitory (*i*, *I*), thalamic reticular (*r*, *R*), thalamic specific relay (*s*, *S*) and external (*x*, *X*). (Figure adapted from [8].)

If we consider the bihemispheric corticothalamic system shown in figure 1 [8,9] and denote left- and right-hemisphere quantities by lower and upper case subscripts, respectively, the various populations have firing rate perturbations *Q*_*a*_ relative to their steady-state levels, where *a* denotes excitatory cortical (*e*, *E*), inhibitory cortical (*i*, *I*), thalamic reticular (*r*, *R*), thalamic relay (*s*, *S*) and external (*x*, *X*) populations. The rate *Q*_*a*_ generates a field *ϕ*_*a*_ of axonal pulses that propagates to other populations, as shown in the figure. Each firing rate perturbation *Q*_*a*_ is then given in terms of the input *Q*_*x*_ from an external population *x* by2.1
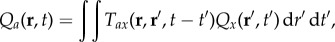
where *T*_*ax*_ is the linear transfer function, or propagator, that relates the activity at **r** and *t* to the input at **r**′ and *t*′, subject to causality (the propagator is zero for *t* < *t*′). External populations are those that never correspond to the first subscript of any transfer function *T*_*ab*_; i.e. that have no feedback to them. If we are concerned with the total time-integrated effect of an input at *t* = 0, we must calculate2.2
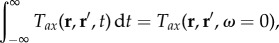
where *ω* is the angular frequency and the quantity on the right of (2.2) is the zero-frequency component of the temporal Fourier transform of *T*_*ax*_.

From this point on, we are only interested in overall effects, not time variations, so we consider only time-integrated transfer functions and spatially averaged activity. In this case, the transfer function to population *a* from population *b* is a real number that does not depend on position, so we omit arguments of transfer functions from here on.

**Figure 2.**
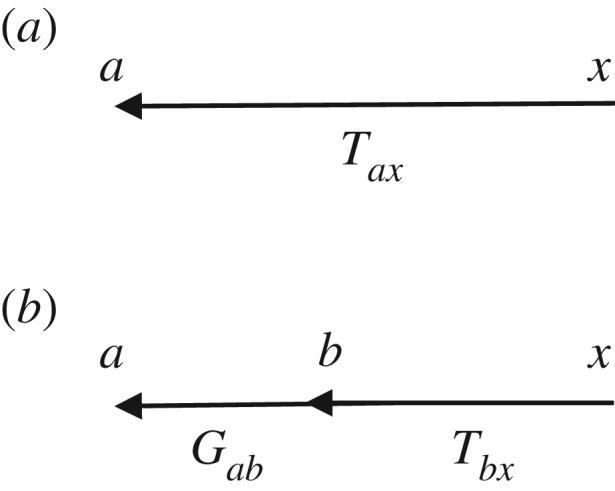
Diagrammatic representation of the propagator and its decomposition. (*a*) Full propagator *T*_*ax*_. (*b*) Decomposition into a sum of terms *G*_*ab*_*T*_*bx*_ to be summed over all populations *b* that project to *a*, including *b* = *a* where there are self-projections.

The transfer function *T*_*ax*_ can be represented diagrammatically by the arrow in figure 2*a*. Robinson showed that this represents a dressed propagator that is the sum total of effects that travel along both direct and indirect paths [5,6]. One can split this propagator into two parts, as shown in figure 2*b* [20]; these represent direct (monosynaptic) propagation to *a* from all directly linked populations *b*, which follows dressed propagation from *x* to the population *b* that contains the final synapse prior to reaching *a*. The direct propagator to *a* from *b* is just the gain *G*_*ab*_, which represents the additional activity in population *a* due to each additional unit of activity afferent from *b* when this link is considered by itself. We thus have2.3
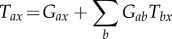
2.4
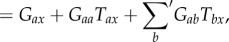
where the sum on the right of (2.3) runs over all internal populations, including *a* itself, but the primed sum in (2.4) excludes population *a*. The splitting in (2.3) can be iterated, leading to an infinite series if there are any feedback loops in the system. Terms arising from loops can be summed to yield2.5
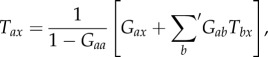
where the *aa* loop has effectively been summed in this case. Note that this result is actually the analytic continuation of the explicit sum, whose convergence would have required |*G*_*aa*_| < 1; it generalizes that result to cases with *G*_*aa*_ ≠ 1. The requirement of system stability, discussed below, places constraints on *T*_*ax*_ that limit individual gains indirectly.

One can interpret the transfer function *T*_*ax*_ as being the additional activity generated at *a* for each additional unit of activity incoming from *x*. The form (2.3) expresses the total transfer of activity to *a* from *x* in terms of a direct gain *G*_*ax*_ plus terms that arise via populations that immediately neighbour *a*. Direct multistep feedforward gains are of the form *G*_*ab*_*G*_*bc*_ … *G*_*zx*_, where there are no intermediate loops.

From equation (2.3), the total magnitude of the influences reaching *a* via various *b* is2.6
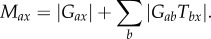
We define the balance parameter2.7
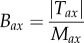
to capture how finely the influences on *a* from *x* are balanced; at perfect balance *B*_*ax*_ = 0.

## 3. Analysis of the bihemispheric corticothalamic system

In previous work, we have applied NFT to the bihemispheric corticothalamic system shown schematically in figure 1 [8]. Here we analyse its large-scale average transfer, balance and criticality properties, as defined in §2.

If we assume symmetry between the hemispheres, we find the following equations for the axonally propagated activity fields *ϕ*_*a*_ generated by population *a* in the low-frequency limit, where all fields are viewed as perturbations from steady-state values:3.1
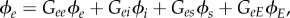
3.2
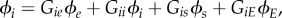
3.3
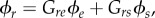
3.4
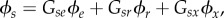
3.5
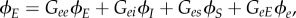
3.6
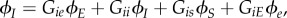
3.7
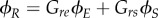
3.8
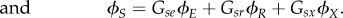
Here all coefficients have been written in terms of left-hemisphere quantities via symmetry. In the case of spatially uniform mean values, as considered here, *ϕ*_*a*_ = *Q*_*a*_ for all populations [2,8].

If we assume the random connectivity approximation that intracortical connections are made with *e* and *i* axonal sources and targets in proportion to the numbers of neurons, we find *G*_*ie*_ = *G*_*ee*_, *G*_*ii*_ = *G*_*ei*_, *G*_*is*_ = *G*_*es*_ and *G*_*iE*_ = *G*_*eE*_ [4,17,19]. Hence, *ϕ*_*i*_ = *ϕ*_*e*_ and *ϕ*_*I*_ = *ϕ*_*E*_. Solution of the above equations then yields3.9
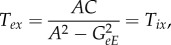
3.10
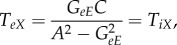
3.11
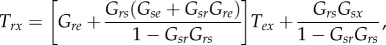
3.12
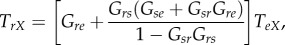
3.13
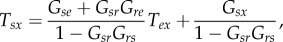
3.14
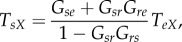
3.15
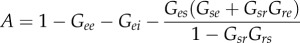
3.16
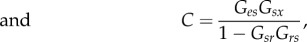
with corresponding equations for the right hemisphere. When there is no interhemispheric coupling, *G*_*eE*_ = 0, *T*_*eX*_ = 0 and *T*_*ex*_ = *C*/*A*, which accords with previous work [2,9].

Instabilities of the system occur when one or other transfer function in (3.9)–(3.14) diverges; i.e. when *G*_*sr*_*G*_*rs*_ = 1 or when *A* ± *G*_*eE*_ = 0. The condition *G*_*sr*_*G*_*rs*_ = 1 cannot be fulfilled for the *ω* = 0 case considered here because *G*_*sr*_ < 0 and *G*_*rs*_ > 0 on physiological grounds. The remaining instability criterion, *A* ± *G*_*eE*_ = 0, can be written as:3.17
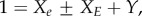
with3.18
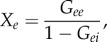
3.19
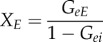
3.20
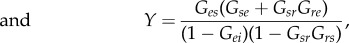
where *Y* is the corticothalamic loop gain [9] and *X*_*e*_ and *X*_*E*_ generalize the corticocortical loop gain of [9] to allow for separate intrahemispheric and interhemispheric contributions, respectively. In (3.17), the upper sign corresponds to *ϕ*_*E*_ = *ϕ*_*e*_ and the lower to *ϕ*_*E*_ = −*ϕ*_*e*_ [8]. These symmetric and antisymmetric solutions are excited by correspondingly symmetric and antisymmetric combinations of inputs with *ϕ*_*X*_ = ±*ϕ*_*x*_, respectively; these can be linearly combined to represent asymmetric inputs. It was recently found that the symmetric solutions have higher *X* + *Y* than antisymmetric ones for *G*_*eE*_ > 0, and are thus are less stable [8], and tend to have higher amplitudes and dominate activity.

The condition *X*_*e*_ + *X*_*E*_ + *Y* = 1 corresponds to criticality, where the least stable mode of the system has unit gain [8]. In (3.17)–(3.20), *X*_*e*_ + *X*_*E*_ + *Y* is the fraction of activity regenerated within the brain, *X*_*e*_ + *Y* is the fraction regenerated within the ipsilateral hemisphere, *X*_*E*_ is the fraction regenerated in the contralateral hemisphere and 1 − *X*_*e*_ − *X*_*E*_ − *Y* is the fraction that enters the corticothalamic system from outside to sustain constant mean activity [4,8,9].

Direct feedforward gains correspond to the paths that lead to a given structure from an external activity source without loops. The resulting gains can be expressed as3.21
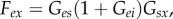
3.22
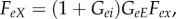
3.23
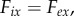
3.24
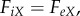
3.25
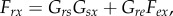
3.26
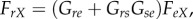
3.27
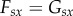
3.28
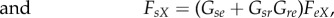
where we have used the random connectivity approximation and symmetry between the hemispheres to simplify these expressions.

## 4. Specific cases

In this section, we examine the balance and criticality of the corticothalamic system for gains obtained from experimental data in our earlier works [1,3,8], allowing for the second hemisphere by splitting the original unihemispheric gains *G*_*ee*_ in a 1 : 5 interhemispheric to intrahemispheric ratio consistent with [6].

Table 1 lists previously obtained representative values of gains *G*_*ab*_ in the unihemispheric corticothalamic system in various states of arousal: eyes open, eyes closed, sleep stages 1 and 2, a sleep-spindle state, slow-wave sleep and REM sleep [1,3].

**Table 1.** Gains *G*_*ab*_ in various states of arousal without allowing explicitly for bihemispheric structure. Here HBM denotes the eyes open state from [1]; the other states are from [3]: eyes open (EO), eyes closed (EC), sleep stage 1 (S1), sleep stage 2 (S2), sleep spindle (S2*σ*), slow wave sleep (SWS) and rapid eye movement sleep (REM). Note that [1] used an estimate *ϕ*_*x*_ = 16 s^−1^, whereas [3] assumed an arbitrary value of *ϕ*_*x*_ = 1 s^−1^ on the grounds that only the product *G*_*sx*_*ϕ*_*x*_ is relevant. This explains the large difference between the values of *G*_*sx*_ in the first two columns; however, *G*_*sx*_*ϕ*_*x*_ is nearly identical in these cases, and the ordering of the other quantities is the same.

state	HBM	EO	EC	S1	S2	S2*σ*	SWS	REM
*G*_*ee*_	6.80	10.5	2.07	7.45	16.86	18.52	19.52	5.87
−*G*_*ei*_	8.10	13.22	4.11	8.30	17.93	18.96	19.74	6.61
*G*_*es*_	1.70	1.21	0.77	0.31	3.89	2.55	5.30	0.21
*G*_*re*_	1.00	0.85	0.66	7.47	4.96	4.67	1.90	2.08
*G*_*rs*_	0.19	0.25	0.20	4.44	8.33	16.92	1.35	4.59
*G*_*se*_	2.50	5.78	7.77	1.67	0.07	0.73	0.22	0.66
−*G*_*sr*_	1.90	2.83	3.30	0.40	0.14	0.26	0.22	0.28
*G*_*sx*_	0.80	14.23	8.10	3.90	2.38	2.78	1.70	0.68

### 4.1. Unihemispheric alert waking state

We begin by considering transfer functions and related quantities for the alert waking state parameters from the column headed HBM in table 1, which omit the bihemispheric structure of the brain.

The second row in table 2 shows the feedforward gains to structures labelled *a* in the first row from equations (3.21)–(3.28), all normalized by dividing by *G*_*sx*_ to remove the effects of differing levels of input. The normalized feedforward to *s* is exactly unity because it only has one input, *G*_*sx*_ itself, as seen in figure 1. The other structures have large negative gains, which arise because *G*_*ei*_*G*_*is*_ + *G*_*es*_ = *G*_*es*_(1 + *G*_*ei*_) is negative, so the net feedforward effect of *s* on *e* (partly via *i*) is negative, and this further propagates to *r*. Hence, in the absence of feedbacks within the cortex (*G*_*ee*_, *G*_*ii*_), thalamus (*G*_*sr*_) and from cortex to thalamic relay nuclei (*G*_*se*_), the net effect of external inputs is highly inhibitory. This strong inhibition may help to explain the existence of coma when certain feedback paths are damaged.

**Table 2.** Transfer functions and related measures for the HBM eyes open state in table 1, normalized where indicated by dividing by *G*_*sx*_ to remove the effects of differing levels of input. The first row lists the structures *a* = *e*, *i*, *r*, *s*. The second row shows the corresponding feedforward gain *F*_*ax*_/*G*_*sx*_ from equations (3.21)–(3.28). The third row shows the total transfer function *T*_*ax*_/*G*_*sx*_, which is the sum of the next five rows aside from numerical round-off error. The final five rows show total gain magnitude *M*_*ax*_/*G*_*sx*_ from (2.6), the balance parameter *B*_*ax*_ from (2.7), *X*_*e*_ from (3.18), *Y* from (3.20) and the criticality parameter *C* from (3.17); the last three parameters apply to all populations equally, and *X*_*E*_ = 0 here.

*a*	*e*	*i*	*r*	*s*
*F*_*ax*_/*G*_*sx*_	−12.1	−12.1	−11.9	1
*T*_*ax*_/*G*_*sx*_	0.81	0.81	1.01	1.09
*G*_*ax*_/*G*_*sx*_	0	0	0	1
*G*_*ae*_*T*_*ex*_/*G*_*sx*_	5.48	5.48	0.81	2.01
*G*_*ai*_*T*_*ix*_/*G*_*sx*_	−6.53	−6.53	0	0
*G*_*ar*_*T*_*rx*_/*G*_*sx*_	0	0	0	−1.92
*G*_*as*_*T*_*sx*_/*G*_*sx*_	1.85	1.85	0.21	0
*M*_*ax*_/*G*_*sx*_	13.9	13.9	1.01	4.93
*B*_*ax*_	0.058	0.058	1.00	0.22
*X*_*e*_	0.75			
*Y*	0.08			
*C*	0.83			

In contrast with pure feedforward gains, the overall normalized transfer functions in the third row of table 2 are all close to unity, indicating that external activity is transferred with little overall amplification or attenuation other than by *G*_*sx*_. This is accomplished by large, near-balanced gains to the cortex, in particular, as seen from the next five rows in table 2, which sum to give the third row. Notably, excitatory and inhibitory cortical gains are almost an order of magnitude larger than the total transfer function to these structures; intrathalamic gains are smaller, especially in the case of *r*, which has only positive inputs in this model (an inhibitory *rr* connection exists but is omitted here because all the gain estimates in prior work were made without including this connection). These findings are reflected in the normalized total transfer magnitudes *M*_*ax*_/*G*_*sx*_ from equation (2.6). Except in the case of *r*, where all input gains have the same sign, these magnitudes are far larger than the individual, near-balanced terms. The degree of balance is seen in the second last row of table 2, which shows that the cortex, in particular, is only a few per cent away from exact balance. This accords with longstanding arguments that near balance makes the system more sensitive to external stimuli, improves precision of neural coding and facilitates complex dynamics [14,15,17].

The final row in table 2 shows that the system is close to the critical point where its fixed point becomes unstable to a saddle–node bifurcation at *C* = 1. The criticality parameter of 0.83 implies that 83% of net activity is regenerated within the brain, 75% within the cortex and 8% via net corticothalamic feedback, while 17% is provided by external inputs, consistent with our prior NFT cortical and corticothalamic models [1,4,9]. More recently, equivalent behaviour was seen in a variety of EEG and fMRI experiments on up to 1500 subjects. In functional connectivity simulations [2,3,5–7,10,19] artificial cell culture experiments saw related avalanche behaviour [12], and other authors have argued for slightly subcritical dynamics on the basis of spiking avalanches observed in animals [13].

### 4.2. Bihemispheric alert waking state

In this section, we explore the effects of the bihemispheric structure on the transfer functions. This is done by dividing the unihemispheric gain *G*_*ee*_ into parts that come from the ipsilateral *G*_*ee*_ and contralateral *G*_*eE*_ hemispheres, keeping the total equal to the original value. We use a 5 : 1 ratio between these two contributions, in accord with the split inferred from eigenfunction analysis of fMRI data [6,8], and assume symmetry of the parameters between the two hemispheres. Table 2 summarizes the resulting transfer measures.

The second row of table 3 shows that intrahemispheric feedforward gains are large, negative (except to *s*, as discussed above) and very similar to those in the unihemispheric case. Feedforward gains from the contralateral hemisphere are even larger, but positive, because they pass through two sets of strong cortical gains, and are twice inverted in sign. Despite the 1 : 5 ratio of interhemispheric to intrahemispheric gains, the interhemispheric feedforward effects would overwhelm intrahemispheric inhibition if only feedforward gains were present. Hence, damage that changes intracerebral gains could drive the brain into either coma or seizure, depending on exactly which connections are affected.

**Table 3.** Normalized transfer functions and related measures for the HBM eyes open state in table 1, with *G*_*ee*_ divided between *G*_*ee*_ and *G*_*eE*_ in a 5 : 1 ratio; entries are normalized where necessary by dividing by *G*_*sx*_ to remove the effects of differing levels of input. The first row lists the structures *a* = *e*, *i*, *r*, *s*. The second and third rows show the corresponding feedforward gains *F*_*ax*_/*G*_*sx*_ and *F*_*aX*_/*G*_*sx*_ from equations (3.21)–(3.28). The fourth and fifth rows show the total transfer functions *T*_*ax*_/*G*_*sx*_, which is the sum of row 6–11 aside from numerical round-off error. The final four row show total gain magnitude *M*_*ax*_/*G*_*sx*_ from (2.6), the balance parameter *B*_*ax*_ from (2.7), *B*_*aX*_ = *T*_*aX*_/*M*_*ax*_ and the criticality parameter from (3.17) which applies to all populations equally.

*a*	*e*	*i*	*r*	*s*
*F*_*ax*_/*G*_*sx*_	−12.1	−12.1	−11.9	1
*F*_*aX*_/*G*_*sx*_	99	99	146	59
*T*_*ax*_/*G*_*sx*_	0.56	0.56	0.75	0.98
*T*_*aX*_/*G*_*sx*_	0.24	0.24	0.26	0.11
*G*_*ax*_/*G*_*sx*_	0	0	0	1
*G*_*ae*_*T*_*ex*_/*G*_*sx*_	3.19	3.19	0.56	1.41
*G*_*aE*_*T*_*Ex*_/*G*_*sx*_	0.28	0.28	0	0
*G*_*ai*_*T*_*ix*_/*G*_*sx*_	−4.57	−4.57	0	0
*G*_*ar*_*T*_*rx*_/*G*_*sx*_	0	0	0	−1.43
*G*_*as*_*T*_*sx*_/*G*_*sx*_	1.67	1.67	0.19	0
*M*_*ax*_/*G*_*sx*_	9.7	9.7	0.75	3.84
*B*_*ax*_	0.058	0.058	1	0.26
*B*_*aX*_	0.025	0.025	0.35	0.029
*X*_*e*_	0.62			
*X*_*E*_	0.13			
*Y*	0.08			
*C*	0.83			

The third and fourth rows in table 3 show that internal feedbacks are able to moderate the huge feedforward gains to produce total transfer functions whose sum is almost identical to those in table 2. However, the effect of feedbacks means that the ratios of the intra- and interhemispheric contributions *T*_*ax*_/*T*_*aX*_ are not 5 : 1; instead 10–30% of input to various populations arises from the contralateral hemisphere, on the assumption of equal external inputs to both hemispheres. Notably, cortical structures have the largest fractional input from the contralateral hemisphere, to which they are directly connected, whereas thalamic ones have less.

The total influences *M*_*ax*_/*G*_*sx*_ and balance parameters *B*_*ax*_ in the second last row of table 3 are similar to the unihemispheric values. The second last row shows *B*_*aX*_ = *T*_*aX*_/*M*_*ax*_, which measures how large an equal contralateral input's influence would be relative to the ipsilateral total. Again, we see that the system is closely balanced with the net transfer function from the ipsilateral hemisphere being only a few per cent of influence on the cortex, while the transfer from the contralateral hemisphere would be only around 2% for the same external input.

The final row in table 3 shows that the stability parameter is unchanged, a result that follows from the way we have partitioned the unihemispheric *G*_*ee*_ into *G*_*ee*_ and *G*_*eE*_. However, we see that the total net influence is 17% external, 8% from corticothalamic feedbacks, 13% from the contralateral hemisphere and 62% from intracortical feedbacks [6].

### 4.3. Comparison of states

In this section, we use gain data for a variety of vigilance states [1,3] to examine and compare the transfer, influence, balance and criticality parameters for conditions ranging from alert waking to deep sleep in both unihemispheric and bihemispheric cases. This has the potential to reveal trends and key differences in the relative orientation of the brain towards internal and external sources of activity. The alert waking data from the previous subsection (HBM) [1] are used, along with data from a more recent study that included alert waking (EO), eyes closed waking (EC), sleep stage 1 (S1), sleep state 2 (S2), slow-wave sleep (SWS), rapid eye movement sleep (REM) and sleep stage 2 with prominent approximately 14 Hz sleep spindles (S2*σ*) [3]. All the gains were inferred from fits of our neural field model to EEG spectra.

Table 4 shows the transfer functions to the cortical excitatory (and inhibitory) population, and the corresponding balance and criticality parameters for a unihemispheric brain, using the parameters from table 1. Key features that are observed, and some comments regarding their potential roles, are as follows. (i) All states are slightly subcritical with *C* = 0.79 − 0.92. (ii) The states furthest from criticality (i.e. the most stable) are S1 and REM. We speculate that S1 has enhanced stability because it results from a sudden reduction of corticothalamic feedback from a positive value in wake to a negative one in sleep, prior to the cortex fully increasing its internal feedback to deeper-sleep values. This temporal ordering tends to protect the brain from instability, which could well result if the order were reversed. (iii) Largely because of being more stable, S1 and REM also have the lowest overall transfer functions, which indicates that they are the least affected at the cortical level by stimuli from the outside world. This may assist in reducing wakenings from these lighter-sleep states and thus help to stabilize the wake-to-sleep transition. (iv) S1 and REM also have the lowest *M*_*ex*_/*G*_*sx*_ and highest *B*_*ex*_ parameters, indicating that they are the furthest from balance but have correspondingly low total input magnitudes to the *e* population. Again, it is possible that a reduction in the sizes of the near-balanced gains makes the system less susceptible to perturbations in light sleep. (v) The deeper sleep states S2 and SWS have the highest transfer functions and smallest balance parameters. This implies that, somewhat paradoxically, they are more influenced by activity that is induced in the relay nuclei than are other states. It is possible that this is an evolutionary adaptation to permit rapid awakening from deep sleep when necessary—small external inputs can produce large changes in cortical activity, and small resulting percentage shifts in individual gains can produce a large net gain. These features are consistent with the fact that evoked potentials in deep sleep—K complexes—are of very large amplitude relative to other normal EEG phenomena [21].

**Table 4.** Unihemispheric cortical transfer functions and related measures for the states in table 1, normalized where necessary by dividing by *G*_*sx*_ to remove the effects of differing levels of input. The first row lists the states from table 1. The second row shows the total transfer function *T*_*ex*_/*G*_*sx*_. The third and fourth rows show total gain magnitude *M*_*ex*_/*G*_*sx*_ from (2.6) and the balance parameter *B*_*ex*_ from (2.7). The final three rows show *X*_*e*_ from (3.18), *Y* from (3.20), and the criticality parameter *C* from (3.17); these apply to all populations equally.

state	HBM	EO	EC	S1	S2	S2*σ*	SWS	REM
*T*_*ex*_/*G*_*sx*_	0.81	0.53	1.04	0.055	0.56	0.28	2.01	0.053
*M*_*ex*_/*G*_*sx*_	13.9	14.6	9.6	0.98	20.7	11.0	82	0.75
*B*_*ex*_	0.058	0.036	0.11	0.057	0.027	0.026	0.025	0.070
*X*_*e*_	0.75	0.74	0.41	0.80	0.89	0.93	0.93	0.77
*Y*	0.08	0.17	0.51	−0.02	−0.06	−0.01	−0.01	0.00
*C*	0.83	0.91	0.91	0.79	0.83	0.92	0.90	0.77

The results in table 5 are the bihemispheric generalizations of the unihemispheric ones in table 4. The results are generally very similar, except for the splitting between hemispheres which leaves total transfer functions unchanged when summed across both hemispheres. Notably, the balance parameter *B*_*eX*_ = *T*_*eX*_/*M*_*ex*_ is only around 1–5%, indicating that contralateral inputs are a very small fraction of the total influence on excitatory (and inhibitory) cortical neurons. The loop gain *Y* and the criticality parameter *C* are unchanged from table 4, while *X*_*e*_ and *X*_*E*_ are simply split in the 5 : 1 ratio that we have assumed in this analysis.

**Table 5.** Bihemispheric transfer functions and related measures for the states in table 1, with the unihemispheric *G*_*ee*_ divided between *G*_*ee*_ and *G*_*eE*_ in a 5 : 1 ratio; entries are normalized where necessary by dividing by *G*_*sx*_ to remove the effects of differing levels of input. The first row lists the states considered. The second and third rows show the total transfer functions *T*_*ex*_/*G*_*sx*_ and *T*_*eX*_/*G*_*sx*_. The next row shows the parameter *M*_*ex*_/*G*_*sx*_ from (2.6). The balance parameter *B*_*ex*_ from (2.7) and *B*_*eX*_ = *T*_*eX*_/*M*_*ex*_ are shown in rows 5 and 6. The final four rows show *X*_*e*_, *X*_*E*_ and *Y* from (3.18) to (3.20), and the criticality parameter *C* from (3.17).

state	HBM	EO	EC	S1	S2	S2*σ*	SWS	REM
*T*_*ex*_/*G*_*sx*_	0.56	0.34	0.72	0.040	0.38	0.17	1.24	0.039
*T*_*eX*_/*G*_*sx*_	0.24	0.19	0.32	0.016	0.18	0.11	0.77	0.014
*M*_*ex*_/*G*_*sx*_	9.7	9.3	6.7	0.71	14.1	6.7	50	0.55
*B*_*ex*_	0.058	0.036	0.11	0.057	0.027	0.026	0.025	0.026
*B*_*eX*_	0.025	0.020	0.049	0.023	0.013	0.017	0.016	0.009
*X*_*e*_	0.62	0.62	0.34	0.67	0.74	0.78	0.78	0.64
*X*_*E*_	0.13	0.12	0.07	0.13	0.15	0.15	0.15	0.13
*Y*	0.08	0.17	0.51	−0.02	−0.06	−0.01	−0.01	0.00
*C*	0.83	0.91	0.91	0.79	0.83	0.92	0.90	0.77

## 5. Summary and discussion

We have used neural field transfer functions to analyse the transfer, stability and balance of large-scale, time-integrated activity into, within and between corticothalamic populations and hemispheres, using gain parameters found for various brain states in prior studies. Key aims were to determine criticality and introspection across states. The main outcomes are:

(i) Time-integrated unihemispheric and bihemispheric NFT transfer functions for each corticothalamic population were derived in terms of gains. These were then used to define total-influence, balance and criticality parameters. In the case in which the brain is treated as a single entity without bihemispheric structure these results reproduced a number of previous analyses.
(ii) Feedforward gains to the cortex are large and negative for intrahemispheric connections, and large and positive for interhemispheric ones, the latter resulting from two successive negative stages. However, overall normalized transfer functions are close to unity, implying that internal feedbacks strongly moderate the feedforward gains to produce little overall amplification or attenuation of activity induced in thalamic relay nuclei by external stimuli. This situation is maintained when the bihemispheric structure of the brain is taken into account.
(iii) Following from point (ii), the total transfer function to the cortex is the sum of large excitatory and inhibitory contributions, balanced to within a few per cent of the total sum of their magnitudes. This means that small percentage changes in the individual gains can make a large fractional change in the total, so the brain is much more rapidly adaptable than if only positive gains existed. For example, to change a positive gain of 0.83 to 0.91 would require a 10% change if it were the result of changing a lone positive gain by this amount. However, if the net gain of 0.83 were the result of two near-balanced gains of order 10 in magnitude, less than a 1% change in either of these gains would be required to achieve the same outcome. This means that rapid changes in brain activity can be achieved via relatively small changes in positive, negative, interhemispheric and/or corticothalamic gains.
(iv) In all brain states investigated, the overall stability parameter lay between 0.77 and 0.92, indicating that they are slightly subcritical. In states other than sleep stage 1 (S1) and REM sleep, the system was finely balanced, to within a few per cent, between positive and negative gains. These states had the lowest net transfer functions and were the furthest from criticality, possibly helping to enhance the stability of wake–sleep transitions. We suggest that these features may reflect a need to reduce waking events in these light-sleep states. Notably, transfer functions were larger in S2 and SWS states, with closer balance and closer proximity to criticality—perhaps enhancing the ability to wake rapidly from these deeper-sleep states in the event of a strong external stimulus.
  An overall implication of criticality and balance is that the brain is extremely agile and can respond rapidly to slight changes in individual near-balanced gains; even small influences from the opposite hemisphere can make a large fractional change to the total. This is particularly important to enable nonlinear effects such as habituation and facilitation to modulate total activity rapidly and effectively without changing synaptic strengths or firing thresholds by large amounts.
(v) In alert waking, cortical activity was found to result approximately 75% from internal feedbacks (split approx. 62% to 13% between intrahemispheric and interhemispheric parts in the bihemispheric case), 8% from net corticothalamic feedbacks, and 17% from external inputs, consistent with an overall criticality parameter of 0.83. Similar results were found for other states. Hence, the brain is highly introspective, and even more so if the net activity is viewed as a fraction of the summed magnitudes of incoming influences, which is of the order of 10–40 times larger, depending on the state.
(vi) Generally, allowing for bihemispheric structure did not change the above qualitative results, although there were quantitative modifications.

Overall, the above results indicate the utility and importance of transfer functions in understanding how influences propagate through the brain. In future, this work should be extended to non-zero frequencies to study the brain from a control-systems perspective. Spatial dependences should also be added to analyse spatially localized criticality, balance and other transfer properties, while time-dependent transfer functions can be expected to yield insights into dynamic processes that underlie cognition and action. Nonetheless, the large-scale average transfer properties discussed here provide the background against which these finer scale dynamics occur. We also note the need to incorporate additional structures and connections such as adding the basal ganglia, dividing short-range cortical neurons into excitatory and inhibitory subtypes, and including a self-connection of the reticular population. In the nonlinear case, seizure propagation between hemispheres is of great interest in clinical settings, thereby underlining the importance of the gain *G*_*eE*_.

## References

[1] Robinson PA, Rennie CJ, Rowe DL, O'Connor SC 2004 Estimation of multiscale neurophysiologic parameters by electroencephalographic means. Hum. Brain Mapp. 23, 53–72. (10.1002/hbm.20032) 15281141 PMC6871818

[2] Robinson PA, Rennie CJ, Rowe DL, O'Connor SC, Gordon E 2005 Multiscale brain modelling. Phil. Trans. R. Soc. B 360, 1043–1050. (10.1098/rstb.2005.1638) 16087447 PMC1854922

[3] Abeysuriya RG, Rennie CJ, Robinson PA 2015 Physiologically based arousal state estimation and dynamics. J. Neurosci. Methods 253, 55–69. (10.1016/j.jneumeth.2015.06.002) 26072247

[4] Robinson PA, Rennie CJ, Wright JJ 1997 Propagation and stability of waves of electrical activity in the cerebral cortex. Phys. Rev. E 56, 826–840. (10.1103/PhysRevE.56.826)

[5] Robinson PA 2012 Interrelating anatomical, effective, and functional brain connectivity using propagators and neural field theory. Phys. Rev. E 85, 011912 (10.1103/PhysRevE.85.011912) 22400596

[6] Robinson PA, Sarkar S, Pandejee GM, Henderson JA 2014 Determination of effective brain connectivity from functional connectivity with application to resting state connectivities. Phys. Rev. E 90, 012707 (10.1103/PhysRevE.90.012707) 25122335

[7] van Albada SJ, Kerr CC, Chiang AKI, Robinson PA 2010 Neurophysiological changes with age probed by inverse modeling of EEG spectra. Clin. Neurophysiol. 121, 21–38. (10.1016/j.clinph.2009.09.021) 19854102

[8] Robinson PA, Zhao X, Aquino KM, Griffiths JD, Sarkar S, Mehta-Pandejee G 2016 Inference of direct and multistep effective connectivities from functional. NeuroImage 142, 79–98. (10.1016/j.neuroimage.2016.04.050) 27157788

[9] Robinson PA, Rennie CJ, Rowe DL 2002 Dynamics of large-scale brain activity in normal arousal states and epileptic seizures. Phys. Rev. E 65, 041924 (10.1103/PhysRevE.65.041924) 12005890

[10] Deco G, Mcintosh AR, Shen K, Hutchison RM, Menon RS, Everling S, Hagmann P, Jirsa VK 2014 Identification of optimal structural connectivity using functional connectivity and neural modeling. J. Neurosci. 34, 7910–7916. (10.1523/JNEUROSCI.4423-13.2014) 24899713 PMC6608269

[11] Haimovici A, Tagliazucchi E, Balenzuela P, Chialvo DR 2013 Brain organization into resting state networks emerges at criticality on a model of the human connectome. Phys. Rev. Lett. 110, 178101 (10.1103/PhysRevLett.110.178101) 23679783

[12] Beggs JM, Plenz D 2003 Neuronal avalanches in neocortical circuits. J. Neurosci. 23, 11167–11177. 14657176 10.1523/JNEUROSCI.23-35-11167.2003 PMC6741045

[13] Priesemann V, Wibral M, Valderrama M, Pröpper R, Le Van Quyen M, Geisel T, Triesch J, Nicolić D, Munk MH 2014 Spike avalanches in vivo suggest a driven, slightly subcritical brain state. Front. Syst. Neurosci. 8, 108 (10.3389/fnsys.2014.00108) 25009473 PMC4068003

[14] Salinas E, Sejnowski TJ 2000 Impact of correlated synaptic input on output firing rate and variability in simple neuronal models. J. Neurosci. 20, 6193–6209. 10934269 10.1523/JNEUROSCI.20-16-06193.2000 PMC6772574

[15] Deneve S, Machens CK 2016 Efficient codes and balanced networks. Nat. Neurosci. 19, 375–382. (10.1038/nn.4243) 26906504

[16] Okun M, Lampl I 2008 Instantaneous correlation of excitation and inhibition during ongoing and sensory-evoked activities. Nat. Neurosci. 11, 535–537. (10.1038/nn.2105) 18376400

[17] Wright JJ, Liley DTJ 1996 Dynamics of the brain at global and microscopic scales: neural networks and the EEG. Behav. Brain Sci. 19, 285 (10.1017/S0140525X00042679)

[18] Robinson PA, et al. 2015 A multiscale working brain model. In Validating computational models in neurological and psychiatric disorders (eds Battacharya BS, Chowdhury FN), ch. 4, p. 107 Cham, Switzerland: Springer.

[19] Deco G, Jirsa VK, Robinson PA, Breakspear M, Friston K 2008 The dynamic brain: from spiking neurons to neural masses and cortical fields. Pub. Lib. Sci. Comp. Biol. 4, e1000092 (10.1371/journal.pcbi.1000092) PMC2519166 18769680

[20] Bjorken JD, Drell SD 1964 Relativistic quantum mechanics, p. 82 New York, NY: McGraw-Hill.

[21] Niedermeyer E, Lopes da Silva FH 1999 Electroencephalography: basic principles, clinical applications, and related fields, p. 174 ff Philadelphia, PA: Lippincott Williams and Wilkins.

